# Extracorporeal Shockwave Therapy Versus Platelet Rich Plasma Injection in Patients of Chronic Plantar Fasciitis: A Randomized Controlled Trial From a Tertiary Center of Eastern India

**DOI:** 10.7759/cureus.34430

**Published:** 2023-01-31

**Authors:** Sanjay Pandey, Niraj Kumar, Anjani Kumar, Anurug Biswas, Upasna Sinha, Jyoti Pandey, Srutarshi Ghosh, Subha Das, Renu A Johnson, Ranjeet Kumar, Anjusha E V, Kalyani Kumari

**Affiliations:** 1 Physical Medicine and Rehabilitation, All India Institute of Medical Sciences, Patna, Patna, IND; 2 Radiology, All India Institute of Medical Sciences, Patna, Patna, IND; 3 Paediatrics, Darbhanga Medical College and Hospital, Darbhanga, IND

**Keywords:** heel and sole pain, plantar fascia thickness, platelet-rich plasma (prp), extracorporeal shockwave therapy, plantar fasciitis (pf)

## Abstract

Introduction

Plantar fasciitis is a degenerative condition of the plantar fascia that leads to heel and sole pain. Physical modalities, physiotherapy, medication, and orthoses have been tried before as treatments. Extracorporeal shockwave therapy (ESWT) and the injection of autologous platelet-rich plasma (PRP) are generally effective in the treatment of plantar fasciitis, which might be resistant to other conservative measures. The present study compares the efficacy of ESWT and PRP injection in respect of symptomatic relief, functional improvement, and change in plantar fascia thickness (PFT).

Methods

Seventy-two patients were enrolled and randomized into two groups. Patients in the first group received ESWT, whereas patients in the second group received PRP injections. Patients were evaluated using the Visual Analog Scale (VAS) and the American Orthopedic Foot and Ankle Society (AOFAS) score, along with PFT measurement (using ultrasonography) before the treatment and at days 15, 30, and 90 after the treatment. The X^2^ test was used to compare qualitative variables, and the paired T-test was used to evaluate quantitative data. Quantitative variables had a normal distribution with a standard deviation, and the significance level was set at P-value=0.05.

Results

On day 0, the mean VAS of the ESWT and PRP groups were 6.44±1.11 and 6.78±1.17, respectively (p=0.237). On day 15, the mean VAS of the ESWT and PRP groups were 4.67±1.45 and 6.67±1.35, respectively (p<0.001). At day 30, the mean VAS of the ESWT and PRP groups were 4.97±1.46 and 4.69±1.39, respectively (p=0.391). On day 90, the mean VAS of the ESWT and PRP groups were 5.47±1.63 and 3.36±0.96 (p<0.001). On day 0, the mean PFTs of the ESWT and PRP groups were 4.73±0.40 and 5.19±0.51, respectively (p<0.001). At day 15, the mean PFT of the ESWT and PRP groups were 4.64±0.46 and 5.11±0.62, respectively (p<0.001) which changed to 4.52±0.53 and 4.40±0.58 at day 30 (p<0.001), and to 4.40±0.50 and 3.82±0.45 at day 90 (p<0.001). The mean AOFAS of the ESWT and PRP groups were 68.39±5.88 and 64.86±8.95 on day 0 (p=0.115), 72.58±6.26 and 67.22±10.47 on day 15 (p=0.115), 73.22±6.92 and 74.72±7.52 on day 30 (p=0.276), and 72.75±7.90 and 81.08±6.01 on day 90, respectively (p<0.001).

Conclusion

Both PRP injection and ESWT are very effective methods to improve pain and cause reduced plantar fascia thickness in patients with chronic plantar fasciitis non-responsive to other conservative measures. PRP injection is more effective at a longer duration as compared to ESWT.

## Introduction

Plantar fasciitis (PF) is one of the leading causes of heel pain in the general population. Although self-limiting, PF may adversely affect physical activity and quality of life [1,2]. In the general community, plantar fasciitis affects about 10% of people [3]. The majority of people are middle-aged, athletes, military personnel, people using hard-sole shoes, and people with sedentary lives. Plantar fasciitis is relatively more common in females [4].

Plantar fasciitis is a non-inflammatory, degenerative condition caused by repeated trauma-generating microtears at the calcaneal enthesis as a result of prolonged standing or jogging. The causes of PF are multifaceted. Weak foot biomechanics, intrinsic muscle weakness, lengthy periods of standing and walking, unsuitable footwear, decreased plantar fascia flexibility, a greater body mass index (BMI), and foot deformities are all potential predisposing factors [1,5-7]. Plantar fasciitis is often diagnosed based on a thorough clinical history and physical examination. The majority of patients experience heel pain and tightness when they first get out of bed in the morning or after sitting for an extended period of time. A sharp stabbing pain is felt while palpating the medial plantar calcaneal area [5,8]. Plantar fasciitis is defined as a thickening of the plantar fascia by more than 4 mm and areas of hypoechogenicity, as well as a blurred edge on ultrasonography (USG) [5]. Lifestyle modifications, ice therapy, non-steroidal anti-inflammatory medicines (NSAIDs), physiotherapy (stretching of the plantar fascia, isometric foot muscle exercises, marble floor exercises, etc.), physical modalities (ultrasound therapy, shockwave therapy, interferential therapy, etc.), shoe modification (soft cushioned heels and soles), and orthotics are all conservative treatment options for plantar fasciitis. Local steroid injections, platelet-rich plasma (PRP), intralesional botulinum toxin A, extracorporeal shockwave therapy (ESWT), and other therapies for plantar fasciitis are also available for chronic disease [7]. Autologous PRP is made from a person's whole blood, which is centrifuged to eliminate red blood cells. The growth factors in the residual plasma are five- to tenfold higher than they are in whole blood. Researchers from various fields have discovered that these growth factors boost natural healing responses [9,10]. Multiple studies have demonstrated that ESWT can destroy sensory unmyelinated nerve fibers, and it can also stimulate neovascularization and collagen synthesis in degenerative tissues, although the exact processes of ESWT in treating musculoskeletal pain remain unknown [11]. Surgery is not common as a treatment for plantar fasciitis [12].

There are fewer studies being done to compare the efficacy of ESWT and PRP for the treatment of chronic plantar fasciitis. In this study, the long-term efficacy of ESWT and PRP was assessed clinically and radiologically. We tried to find out which one was more efficacious between USG-guided PRP injection and ESWT for the treatment of chronic plantar fasciitis.

## Materials and methods

**Figure 1:**
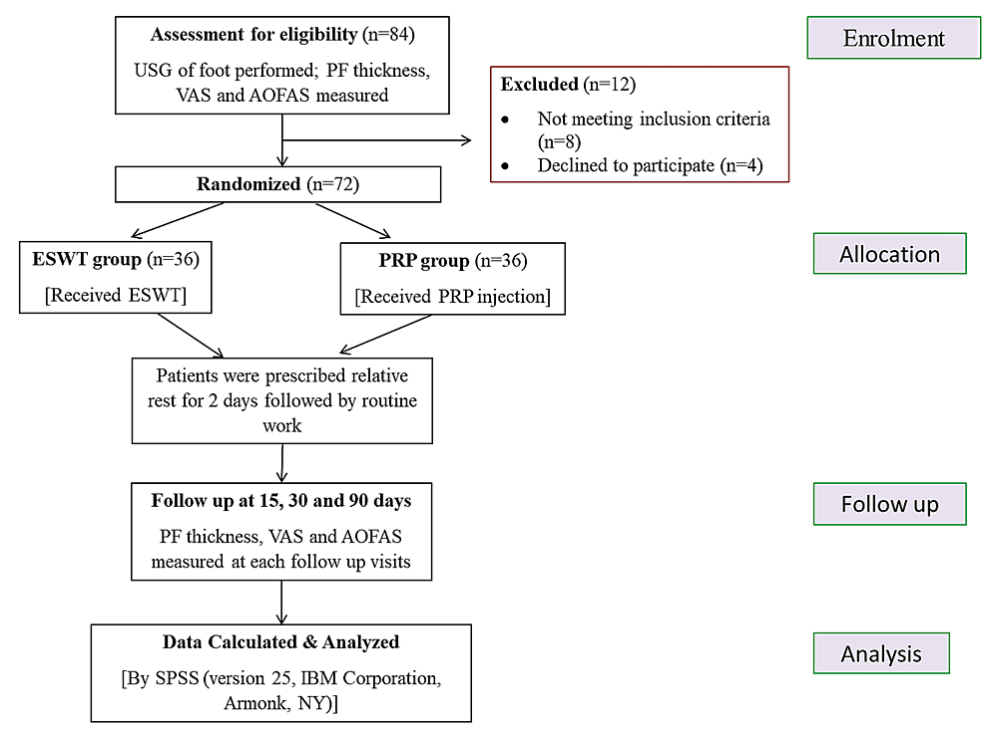
CONSORT diagram CONSORT: Consolidated Standards of Reporting Trials; USG: ultrasonography; VAS: Visual Analog Scale; PF: plantar fascia; ESWT: extracorporeal shockwave therapy; PRP: platelet rich plasma; AOFAS: American Orthopedic Foot and Ankle Society.

This study aimed to compare the effectiveness of autologous PRP injection and extracorporeal shockwave therapy in treating chronic plantar fasciitis. This randomized controlled clinical trial was done at the Department of Physical Medicine and Rehabilitation in a tertiary care center in eastern India. The study period was from April 2021 to June 2022. Based on inclusion and exclusion criteria, a total of 72 individuals with chronic plantar fasciitis for more than two months [13] who had not responded to conservative treatments such as physical therapy (stretching of the plantar fascia, isometric foot muscle exercises, cold therapy, etc.), NSAIDs, and heel cushions and who attended our OPD were enrolled. Written informed consent was obtained from all the participants. All of the patients were thoroughly examined. Patients were randomized into two groups by using online software. Allocation concealment was done. Blinding was not feasible because the patient knew the treatment, as one intervention, was injectable. However, the outcome measure was assessed by another person who was not aware of the treatment the patients had received, minimizing information bias. The patients in the first group were given ESWT treatments, whereas the patients in the second group were given PRP injections. All participants were advised to stop taking NSAIDs three days prior to the procedure. The Consolidated Standards of Reporting Trials (CONSORT) guideline was followed properly (Figure 1).

Ethical clearance and trial registration

Patient enrollment began after approval by the institutional research committee and the institutional ethics committee (ref. no. AIIMS/Pat/IEC/PGTh/Jan20/29). The study is registered with a clinical trial registry under the number CTRI/2021/04/033178.

Inclusion criteria

Consenting patients with chronic plantar fasciitis, aged between 18 and 65 years, and with pain intensity greater than ≥5 on the VAS were included in the study.

Exclusion criteria

Patients with a cardiac pacemaker, a calcaneal fracture, previous foot surgery, an autoimmune disorder, a systemic inflammatory disorder, a neurological problem, any psychological disorder, any uncontrolled systemic disorders, bleeding disorder or on anticoagulant therapy, severe anemia and skin infection at the site of injection were excluded from the study.

Sample size calculation

On the basis of the study by Aykol and Ersoy [3], the sample size at a 5% level of significance and 80% power considering a 10% dropout was calculated to be 72.

Methodology

Patients with chronic plantar fasciitis were separated into two groups, each consisting of 36 patients. The ESWT group received shock wave therapy, and the PRP group received an autologous PRP injection. 

Outcome Measurement

Patients were evaluated using a VAS, American Orthopedic Foot and Ankle Society (AOFAS) score, and plantar fascia thickness (PFT) measurement using USG before and at follow-up after the procedures.

The VAS scale is a subjective, validated assessment scale for acute and chronic pain. Scores are measured on a 10-cm VAS scale line and are graded on a scale of 0 (no pain) to 10 (worst pain) [14]. The AOFAS score is a nine-question questionnaire used to assess pain, function, and foot alignment in the foot. It has a total score of 100. A score of 0-69 indicates poor performance, 70-79 indicates moderate performance, 80-89 indicates fair performance, and 90-100 indicates exceptional performance [15].

A single person performed the plantar fascia examinations on the plantar region of the foot using ultrasonography (Sonosite Edge II ultrasound machine). Before the treatment and 15, 30, and 90 days later, PFT was assessed. For the patient's and clinician's convenience, the patient was advised to lie supine/prone with the leg rotated externally. The USG with a high-frequency linear transducer was used to find the area with plantar fascia thickness and changed echogenicity. At 1 cm from the site of the greatest acoustic shadow, which corresponded to the calcaneal tubercle, a transverse measurement in millimeters was taken on the plantar fascia.

Intervention

The patients in the first group underwent ESWT (HC SWT MODEL, Electronica Pagani, Roland Healthcare). The patient was asked to lie down on the examination table without footwear in the supine position. The maximum tender point was identified and marked, and a succession of 2000 shock wave pulses fired at a repetition frequency of two pulses per second was used to treat the affected tissue region. The energy level or intensity was set to an acceptable level (0.2 mJ/mm^2^) [13]. The entire therapy took 15 minutes per session and was done without the use of local anesthetics. Following ESWT administration, the patient was advised to rest for 30 minutes before returning to normal activities.

PRP injections were used in the second group. A total of 20 ml of blood was drawn from the basilic or antecubital veins and transferred to vacuum-sterilized test tubes with sodium citrate as an anticoagulant. Blood samples were centrifuged at 1,200 rotations per minute (rpm) for 12 minutes (Eppendorf Centrifuge 5702). The blood was separated into three layers: an upper layer containing platelets and white blood cells, an intermediate thin layer containing white blood cells (the buffy coat), and a bottom layer containing red blood cells. An empty sterile tube was used to transfer the upper and intermediate buffy layers. The plasma was centrifuged again for seven minutes at 3,300 rpm to aid in the formation of soft pellets (erythrocytes and platelets) at the bottom of the tube; the upper two-thirds of the plasma were discarded due to platelet-poor plasma; pellets were homogenized in the lower third (3 ml) of the plasma to create the PRP; a 5 ml syringe was used to extract the PRP [16].

For the patient's and clinician's convenience, the patient was advised to lie supine/prone with the leg rotated externally. The USG with a high-frequency (6-15 MHz) linear transducer was used to find the area with the most plantar fascia thickness and changed echogenicity. Lignocaine was used as a local anesthetic. After decontamination with betadine solution, PRP was injected using an intramuscular needle (21 gauge). Patients were told not to put any weight on their heels for two days after receiving PRP injections. Patients were advised to use a cold pack twice daily for two days following the PRP injection. They were also advised to wear athletic footwear throughout this period and were not permitted to take NSAIDs.

Follow-Up and Data Analysis

Patients were followed up on the 15th, 30th, and 90th days following the interventions, and their VAS, AOFAS score, and plantar fascia thickness by USG were noted at each follow-up visit. SPSS software was used to examine the data (version 25, IBM Corporation, Armonk, NY). The X^2^ test was used to compare qualitative variables, and the paired T-test was used to evaluate quantitative data. Quantitative variables had a normal distribution with a standard deviation, and the significance level was set at P-value=0.05.

## Results

**Figure 2:**
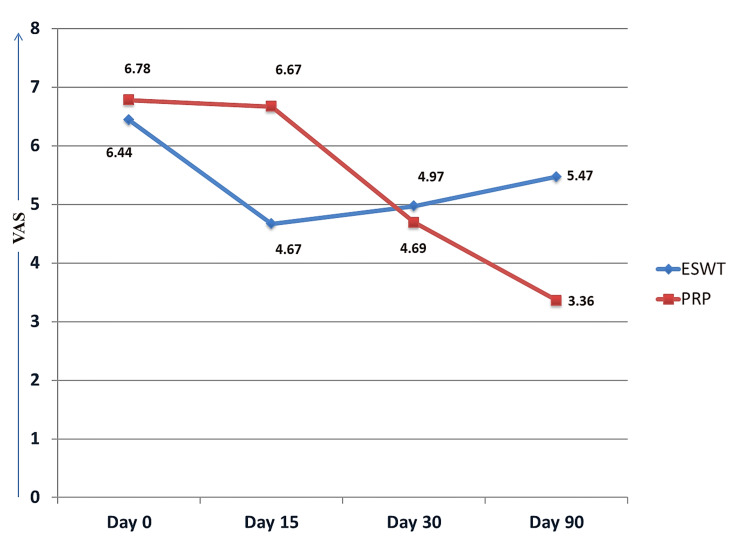
Intergroup and intragroup comparison of VAS VAS: Visual Analog Scale; ESWT: extracorporeal shockwave therapy; PRP: platelet-rich plasma. Note: This figure shows improvement in both ESWT and PRP groups at follow up, more with PRP in longer duration.

The mean age of the ESWT group was 39.61±8.83 years, while the mean age of the PRP group was 38.03±9.96 years. No significant difference was found in mean ages between the groups (p=0.478). The male-female proportion in the study was 40.3 (n=29)/59.7 (n=43). In the ESWT group, this proportion was 33.3 (n=12)/66.7 (n=24), while in the PRP group, the proportion was 47.2 (n=17)/52.8 (n=19). No significant difference was found in the male-female proportion between the groups (p=0.230). The VAS of the ESWT group and the PRP group were compared on days 0, 15, 30, and 90. A significant difference was found in mean VAS between the groups on days 15 and 90 (Figure 2, Table 1).

**Table 1:** Intergroup and intragroup comparison of VAS VAS: Visual Analog Scale; ESWT: extracorporeal shockwave therapy; PRP: platelet rich plasma; SD: standard deviation. p<0.05 is significant.

VAS	ESWT		PRP		Mann Whitney test	
	Mean	SD	Mean	SD	z-value	p-value
0 day	6.44	1.11	6.78	1.17	−1.18	0.237
15 day	4.67	1.45	6.67	1.35	−4.99	<0.001
30 day	4.97	1.46	4.69	1.39	−0.86	0.391
90 day	5.47	1.63	3.36	0.96	−5.31	<0.001
Intragroup (Friedmann test)	Chi-square=40.64, p<0.001		Chi-square=86.71, p<0.001			

**Figure 3:**
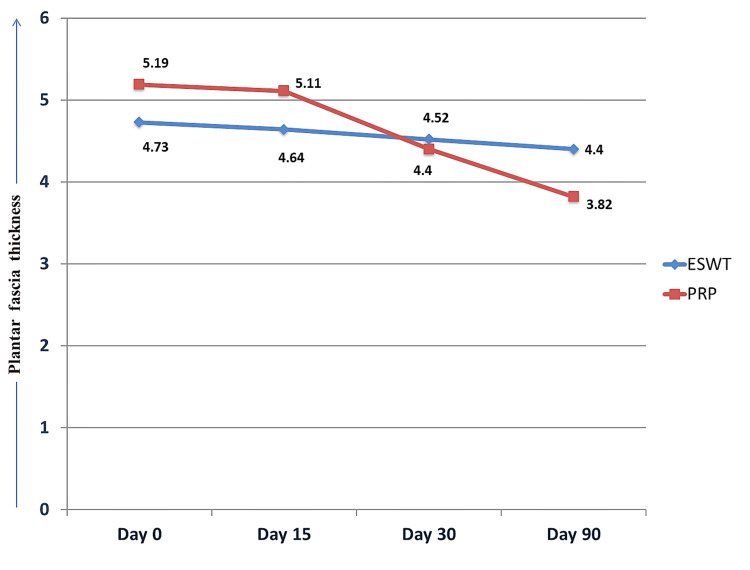
Intergroup and intragroup comparison of plantar fascia thickness ESWT: extracorporeal shockwave therapy; PRP: platelet-rich plasma.

The PFT of the ESWT group and the PRP group were compared on days 0, 15, 30, and 90. A significant difference was found in the mean PFT between the groups on days 0, 15, and 90 (Figure 3, Table 2).

**Table 2:** Intergroup and intragroup comparison of PFT PFT: plantar fascia thickness; ESWT: extracorporeal shockwave therapy; PRP: platelet rich plasma; SD: standard deviation. p<0.05 is significant.

PFT	ESWT		PRP		Mann Whitney test	
	Mean	SD	Mean	SD	z-value	p-value
0 day	4.73	0.40	5.19	0.51	−3.86	<0.001
15 day	4.64	0.46	5.11	0.62	−3.58	<0.001
30 day	4.52	0.53	4.40	0.58	−0.80	0.423
90 day	4.40	0.50	3.82	0.45	−4.45	<0.001
Intragroup (Friedmann test)	Chi-square=27.65, p<0.001		Chi-square=81.76, p<0.001			

**Figure 4:**
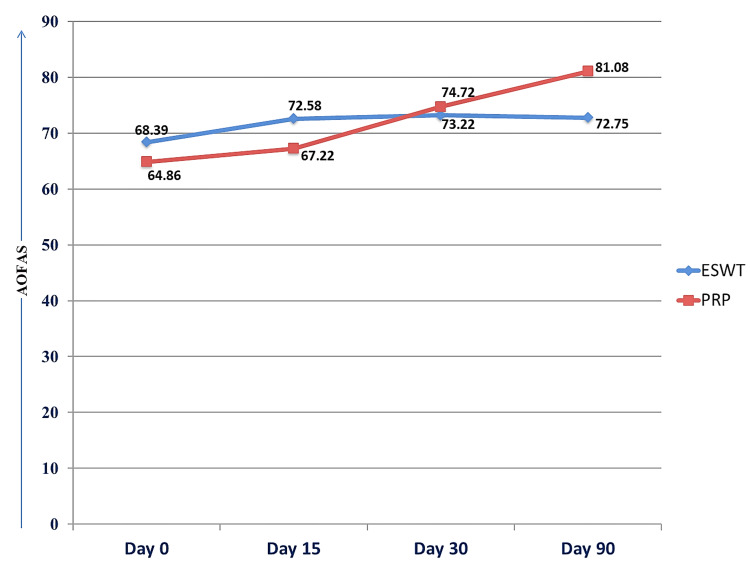
Intergroup and intragroup comparison of AOFAS AOFAS: American Orthopedic Foot and Ankle Society; ESWT: extracorporeal shockwave therapy; PRP: platelet-rich plasma.

AOFAS of the ESWT group and PRP group were compared on days 0, 15, 30, and 90. A significant difference was found in mean AOFAS between the groups on days 15 and 90 (Figure 4, Table 3).

**Table 3:** Intergroup and intragroup comparison of AOFAS AOFAS: American Orthopedic Foot and Ankle Society; ESWT: extracorporeal shockwave therapy; PRP: platelet rich plasma; SD: standard deviation. p<0.05 is significant.

AOFAS score	ESWT		PRP		Mann Whitney test	
	Mean	SD	Mean	SD	z-value	p-value
0 day	68.39	5.88	64.86	8.95	−1.58	0.115
15 day	72.58	6.26	67.22	10.47	−2.40	0.016
30 day	73.22	6.92	74.72	7.52	−1.09	0.276
90 day	72.75	7.90	81.08	6.01	−4.34	<0.001
Intragroup (Friedmann test)	Chi-square=25.55, p<0.001		Chi-square=84.37, p<0.001			

The mean VAS% change of the ESWT group was 15.26±20.27%, while for the PRP group, it was 49.06±16.77%. A significant difference was found in the mean VAS% change between the groups (p<0.001). This change was comparatively more in the PRP group. The mean PFT% change of the ESWT group was 6.80±8.07%, while the mean PFT% change of the PRP group was 25.71±11.33%. A significant difference was found in the mean PFT% change between the groups (p<0.001). This change was comparatively more in the PRP group. The mean AOFAS% change of the ESWT group was 6.52±9.43%, while the mean AOFAS% change of the PRP group was 27.36±19.91%. A significant difference was found in the mean AOFAS% change between the groups (p<0.001). This change was comparatively more in the PRP group (Table 4).

**Table 4:** Intergroup comparison of VAS%, PFT%, and AOFAS% changes VAS: Visual Analog Scale; PFT: plantar fascia thickness; AOFAS: American Orthopedic Foot and Ankle Society; ESWT: extracorporeal shockwave therapy; PRP: platelet rich plasma; SD: standard deviation. p<0.05 is significant.

	ESWT		PRP		Unpaired t test	
	Mean	SD	Mean	SD	t-value	p-value
VAS% change	15.26	20.27	49.06	16.77	−7.71	<0.001
PFT% change	6.80	8.07	25.71	11.33	−8.16	<0.001
AOFAS% change	6.52	9.43	27.36	19.91	−5.68	<0.001

The multivariate analysis showing relationship of VAS%, PFT%, and AOFAS% changes with independent explanatory variables (Table 5).

**Table 5:** Multivariate analysis showing relationship of VAS%, PFT%, and AOFAS% changes with independent explanatory variables VAS: Visual Analog Scale; PFT: plantar fascia thickness; AOFAS: American Orthopedic Foot and Ankle Society; ESWT: extracorporeal shockwave therapy; PRP: platelet rich plasma; B: beta co-efficient; SE: standard error. p<0.05 is significant.

Dependent variable	Explanatory variable	B	SE	t	p-value	Effect size
VAS% change	Intercept	47.07	9.89	4.76	<0.001	0.253
	Age	0.07	0.24	0.30	0.768	0.001
	Male	−1.60	6.39	−0.25	0.804	0.001
	Female	Ref.				
	ESWT	−35.64	5.91	−6.03	<0.001	0.352
	PRP	Ref.				
PFT% change	Intercept	17.97	4.99	3.60	0.001	0.162
	Age	0.11	0.12	0.89	0.376	0.012
	Male	7.55	3.22	2.34	0.022	0.076
	Female	Ref.				
	ESWT	−15.77	2.98	−5.29	<0.001	0.295
	PRP	Ref.				
AOFAS% change	Intercept	24.51	8.23	2.98	0.004	0.117
	Age	0.02	0.20	0.10	0.922	0.000
	Male	4.42	5.31	0.83	0.408	0.010
	Female	Ref.				
	ESWT	−20.02	4.91	−4.07	<0.001	0.199
	PRP	Ref.				

## Discussion

Plantar fasciitis is one of the most common primary causes of heel pain. It is typically a self-limiting disorder, and non-operative treatment is effective in most cases. In this study, there was no statistically significant difference between the baseline parameters of the group that received ESWT and the other group that received PRP. The groups were matched for age and gender.

In the current study, we found that both PRP and ESWT treatments considerably improved the patient's pain, plantar fascia thickness, and AOFAS score. We also demonstrated that individuals receiving PRP treatment experienced more pain relief, a greater reduction in plantar fascia thickness, and a greater improvement in their AOFAS score than those receiving ESWT treatment for a longer duration. The results of the current study demonstrated that there was a significant difference between PRP and ESWT treatment when comparing the mean VAS% change, mean PFT% change, and mean AOFAS% change.

PRP injections at the tender area with or without USG guidance do not differ significantly from one another, according to a study by Kane et al. [17]. ESWT was given at the maximum tender point over the plantar fascia. A succession of 2000 shock wave pulses fired at a repetition frequency of two pulses per second was used [13].

Seventy patients with chronic plantar fasciitis were managed with ESWT in a study by Dastgir in Ireland in 2004, and they found that the patient's pain significantly decreased [18]. The use of ESWT is associated with positive outcomes and pain reductions in patients with chronic plantar fasciitis unresponsive to conventional conservative therapies, according to a meta-analysis of a total of 716 patients by Zhiyun et al. published in 2013 [19]. These results confirmed our findings by demonstrating the high effectiveness of ESWT in treating chronic plantar fasciitis. Many treatment modalities have been in practice, among which ESWT has been extensively used as a treatment option for PF for decades due to its noninvasive nature, fast recovery time, and convenience for the daily lives of patients [20,21]. The actual processes by which it relieves musculoskeletal pain are still unknown. However, various studies show that ESWT may be able to destroy sensory unmyelinated nerve fibers, promote neovascularization, and increase the production of collagen in degenerative tissues [11].

Kalia et al. did a prospective study to determine the role of PRP in chronic plantar fasciitis and observed clinically and statistically significant improvements in VAS scores for heel pain, functional outcome scores in view of the AOFAS score, and reduction of plantar fascia thickness after receiving a single dose of PRP injections [22]. This study concluded that local PRP injections are an effective treatment for chronic plantar fasciitis. These outcomes were comparable with our research. A lot of studies have also demonstrated the efficacy of platelet-rich plasma injection as a therapy for chronic plantar fasciitis [23,24]. PRP has an abundant quantity of growth factors, including vascular endothelial growth factor, transforming growth factor, platelet-derived growth factor, and inflammatory mediators like interleukins and cytokines. There is a low concentration of all such components in the plantar fascia due to hypovascularity and hypocellularity. Local injection of PRP supplies these growth factors and platelets directly to the site of the lesion, which is required in the later stages of healing [25].

Data from 10 clinical trials and 604 individuals with chronic plantar fasciitis were evaluated in another meta-analysis by Hsiao et al. in 2015 [26]. They found that the most successful therapeutic approach was the administration of autologous blood-derived products (ABPs) following corticosteroid injections. They further stated that PRP, a subgroup of ABPs, was thought to be more effective than ESWT and that it was as valuable and efficient as ABPs in terms of causing pain improvements [26]. Here, we can conclude that for acute pain, ESWT works better than PRP. On day 30, both modalities were having an almost similar effect on reducing pain. However, on day 90, the PRP group showed better improvement in VAS and AOFAS scores than the ESWT group. This shows that PRP was better compared to ESWT in controlling pain for a longer duration.

In our study, the patients enrolled for PRP had thicker plantar fascia compared to those enrolled for ESWT. On day 15, the mean PFT of the ESWT group was less than the mean PFT of the PRP group. No significant difference was found in the mean PFT between the groups at day 30. At day 90, the mean PFT of the ESWT group was higher than the mean PFT of the PRP group. Though PFT decreases in both groups significantly, it is concluded that the plantar fascia thickness in the patients of the PRP group decreases more in the long term compared to the ESWT group. In this current study, no major adverse effect was seen in both the PRP and ESWT groups, assuring the safety profile.

Limitation

The limitation of this study was that no blinding of the participants was done. As a result, there could be patient-related bias. However, patients were not given choices for selecting their treatment group. Moreover, the assessor was blinded to minimize the bias. The role of comorbidities on the outcome was not assessed in this study. A longer follow-up is also needed to know the long-term benefits. ESWT is usually applied multiple times, but we only applied it once, which might have affected its long-term outcome in later follow-ups. More studies on similar topics with larger sample sizes are needed for this hour.

## Conclusions

Both PRP injection and ESWT are very effective methods to improve pain and reduce plantar fascia thickness in patients with chronic plantar fasciitis nonresponsive to other conservative measures. The ESWT group showed good initial benefit over the PRP group, but the effect hit a plateau after a couple of months, whereas the PRP group showed improvement after a few weeks from the injection. PRP injection is more effective over a longer duration, though the actual length or duration of its effect is not clear from our study. Both modalities have very good safety profiles and patient compliance. The number of sessions of ESWT and PRP can be altered and studied in the future to find out the algorithms of these interventions that provide a better outcome. Studies combining both methods are also needed in the future.

## References

[1] Gupta AK, Gogia VS, Asthana SS (2018). Association of plantar fasciopathy with plantar fascia thickness: an observational study. Indian J Phys Med Rehabil.

[2] Ehrmann C, Maier M, Mengiardi B, Pfirrmann CW, Sutter R (2014). Calcaneal attachment of the plantar fascia: MR findings in asymptomatic volunteers. Radiology.

[3] Aykol G, Ersoy Y (2016). An evaluation of the efficacy of ESWT and placebo-ESWT treatment for chronic plantar fasciitis. Med Sci.

[4] Rompe JD, Decking J, Schoellner C, Nafe B (2003). Shock wave application for chronic plantar fasciitis in running athletes. A prospective, randomized, placebo-controlled trial. Am J Sports Med.

[5] Karabay N, Toros T, Hurel C (2007). Ultrasonographic evaluation in plantar fasciitis. J Foot Ankle Surg.

[6] Goff JD, Crawford R (2011). Diagnosis and treatment of plantar fasciitis. Am Fam Phys.

[7] Zhao J, Luo WM, Li T (2020). Extracorporeal shock wave therapy versus corticosteroid injection for chronic plantar fasciitis: a protocol of randomized controlled trial. Medicine (Baltimore).

[8] Thomas JL, Christensen JC, Kravitz SR (2010). The diagnosis and treatment of heel pain: a clinical practice guideline-revision 2010. J Foot Ankle Surg.

[9] Lai LP, Stitik TP, Foye PM, Georgy JS, Patibanda V, Chen B (2015). Use of platelet-rich plasma in intra-articular knee injections for osteoarthritis: a systematic review. PM R.

[10] Everts PA, Hoogbergen MM, Weber TA, Devilee RJ, van Monftort G, de Hingh IH (2012). Is the use of autologous platelet-rich plasma gels in gynecologic, cardiac, and general, reconstructive surgery beneficial?. Curr Pharm Biotechnol.

[11] Hsu RW, Hsu WH, Tai CL, Lee KF (2004). Effect of shock‐wave therapy on patellar tendinopathy in a rabbit model. J Orthop Res.

[12] Leão RG, Azuma MM, Ambrosio GH, Faloppa F, Takimoto ES, Tamaoki MJ (2020). Effectiveness of shockwave therapy in the treatment of plantar fasciitis. Acta Ortop Bras.

[13] Eslamian F, Shakouri SK, Jahanjoo F, Hajialiloo M, Notghi F (2016). Extra corporeal shock wave therapy versus local corticosteroid injection in the treatment of chronic plantar fasciitis, a single blinded randomized clinical trial. Pain Med.

[14] Hawker GA, Mian S, Kendzerska T, French M (2011). Measures of adult pain: Visual Analog Scale for Pain (VAS Pain), Numeric Rating Scale for Pain (NRS Pain), McGill Pain Questionnaire (MPQ), Short-Form McGill Pain Questionnaire (SF-MPQ), Chronic Pain Grade Scale (CPGS), Short Form-36 Bodily Pain Scale (SF-36 BPS), and Measure of Intermittent and Constant Osteoarthritis Pain (ICOAP). Arthritis Care Res (Hoboken).

[15] Analay Akbaba Y, Celik D, Ogut RT (2016). ranslation, cross-cultural adaptation, reliability, and validity of Turkish version of the American Orthopaedic Foot and Ankle Society Ankle-Hindfoot Scale. J Foot Ankle Surg.

[16] Dawood AS, Salem HA (2018). Current clinical applications of platelet-rich plasma in various gynecological disorders: an appraisal of theory and practice. Clin Exp Reprod Med.

[17] Kane D, Greaney T, Shanahan M, Duffy G, Bresnihan B, Gibney R, FitzGerald O (2001). The role of ultrasonography in the diagnosis and management of idiopathic plantar fasciitis. Rheumatology (Oxford).

[18] Dastgir N (2014). Extracorporeal shock wave therapy for treatment of plantar fasciitis. J Pak Med Assoc.

[19] Zhiyun L, Tao J, Zengwu S (2013). Meta-analysis of high-energy extracorporeal shock wave therapy in recalcitrant plantar fasciitis. Swiss Med Wkly.

[20] Theodore GH, Buch M, Amendola A, Bachmann C, Fleming LL, Zingas C (2004). Extracorporeal shock wave therapy for the treatment of plantar fasciitis. Foot Ankle Int.

[21] Ogden JA, Alvarez R, Levitt R, Cross GL, Marlow M (2001). Shock wave therapy for chronic proximal plantar fasciitis. Clin Orthop Relat Res.

[22] Kalia RB, Singh V, Chowdhury N, Jain A, Singh SK, Das L (2021). Role of platelet rich plasma in chronic plantar fasciitis: a prospective study. Indian J Orthop.

[23] Monto RR (2014). Platelet-rich plasma efficacy versus corticosteroid injection treatment for chronic severe plantar fasciitis. Foot Ankle Int.

[24] Shetty VD, Dhillon M, Hegde C, Jagtap P, Shetty S (2014). A study to compare the efficacy of corticosteroid therapy with platelet-rich plasma therapy in recalcitrant plantar fasciitis: a preliminary report. Foot Ankle Surg.

[25] Yang WY, Han YH, Cao XW, Pan JK, Zeng LF, Lin JT, Liu J (2017). Platelet-rich plasma as a treatment for plantar fasciitis: a meta-analysis of randomized controlled trials. Medicine (Baltimore).

[26] Hsiao MY, Hung CY, Chang KV, Chien KL, Tu YK, Wang TG (2015). Comparative effectiveness of autologous blood-derived products, shock-wave therapy and corticosteroids for treatment of plantar fasciitis: a network meta-analysis. Rheumatology (Oxford).

